# Flammer syndrome

**DOI:** 10.1186/1878-5085-5-11

**Published:** 2014-07-08

**Authors:** Katarzyna Konieczka, Robert Ritch, Carlo Enrico Traverso, Dong Myung Kim, Michael Scott Kook, Augusto Gallino, Olga Golubnitschaja, Carl Erb, Herbert A Reitsamer, Teruyo Kida, Natalia Kurysheva, Ke Yao

**Affiliations:** 1 Department of Ophthalmology, University of Basel, Mittlere Strasse 91, 4031 Basel, Switzerland; 2 Einhorn Clinical Research Center, New York Eye and Ear Infirmary of Mount Sinai, New York, NY 10003, USA; 3 Clinica Oculistica, Di.N.O.G.M.I., University of Genova, 16132 Genova, Italy; 4 Department of Ophthalmology, Seoul National University, Seoul 110-744, Korea; 5 Department of Ophthalmology, University of Ulsan, Seoul 680-749, Korea; 6 Department of Internal Medicine and Cardiology, Ospedale San Giovanni, 6500 Bellinzona, Switzerland; 7 Department of Radiology, Friedrich-Wilhelms-University of Bonn, 53113 Bonn, Germany; 8 Eye Clinic Wittenbergplatz, 10789 Berlin, Germany; 9 Department of Ophthalmology, SALK/Paracelsus Medical University, 5020 Salzburg, Austria; 10 Department of Ophthalmology, Osaka Medical College, Takatsuki 569-8686, Japan; 11 Department of Diagnostic and Glaucoma, University of Medical and Biological Agency of the Russian Federation, 105077 Moscow, Russian Federation; 12 Eye Center of Second Affiliated Hospital, Zhejiang University College of Medicine, Hangzhou 310009, China

**Keywords:** Flammer syndrome, Primary vascular dysregulation, Vasospasm, Cold extremities, Systemic hypotension, Normal tension glaucoma, Tinnitus, Optic disc hemorrhages, Retinal vein occlusion, Predictive preventive personalized medicine

## Abstract

The new term Flammer syndrome describes a phenotype characterized by the presence of primary vascular dysregulation together with a cluster of symptoms and signs that may occur in healthy people as well as people with disease. Typically, the blood vessels of the subjects with Flammer syndrome react differently to a number of stimuli, such as cold and physical or emotional stress. Nearly all organs, particularly the eye, can be involved. Although the syndrome has some advantages, such as protection against the development of atherosclerosis, Flammer syndrome also contributes to certain diseases, such as normal tension glaucoma. The syndrome occurs more often in women than in men, in slender people than in obese subjects, in people with indoor rather than outdoor jobs, and in academics than in blue collar workers. Affected subjects tend to have cold extremities, low blood pressure, prolonged sleep onset time, shifted circadian rhythm, reduced feeling of thirst, altered drug sensitivity, and increased general sensitivity, including pain sensitivity. The plasma level of endothelin-1 is slightly increased, and the gene expression in lymphocytes is changed. In the eye, the retinal vessels are stiffer and their spatial variability larger; the autoregulation of ocular blood flow is decreased. Glaucoma patients with Flammer syndrome have an increased frequency of the following: optic disc hemorrhages, activated retinal astrocytes, elevated retinal venous pressure, optic nerve compartmentalization, fluctuating diffuse visual field defects, and elevated oxidative stress. Further research should lead to a more concise definition, a precise diagnosis, and tools for recognizing people at risk. This may ultimately lead to more efficient and more personalized treatment.

## Review

### Introduction

Flammer syndrome [1-4] is a cluster of symptoms and signs that can occur in healthy people and people with disease. Its medical relevance lies in the fact that these subjects react differently to a number of stimuli, such as cold [5] and physical or emotional stress and that this aggravated response can induce diseases [6,7]. The term Flammer syndrome is a medical eponym named after the Swiss physician Josef Flammer in recognition of his contribution in this field and of his observations leading to the definition of such a phenotype.

The purpose of this position paper is to summarize current knowledge about the syndrome and to outline future directions for research. The goal is not a copious description of details, but instead a concise overview of Flammer syndrome. For more detailed information on primary vascular dysregulation, including references for original publications, we refer to recent comprehensive reviews [6,7]. The basic aspects of blood flow particularly in the eye are described in textbooks [8-10].

### Terminology

The term Flammer syndrome complements and partly replaces other historical notions briefly described here. *Vasospasm* represents reversible disproportionate constriction of arteries that causes a temporary shortage of blood supply in the corresponding organ or a part of it. Vasospasm has been known in medicine for decades, e.g. in the retina, particularly in the context of migraine. Spasm in diseased vessels or after organ transplantations are not discussed here. Spasm also can occur in morphologically healthy vessels. If spasm is present in several organs of the same subject simultaneously or sequentially, the term *vasospastic syndrome*[5] is used.

With the advent of better technology for analyzing the vascular system, it became clear that arterial vasospasm is only one component of a more general type of dysregulation of arteries, veins, and capillaries. The term *vascular dysregulation*[11] encompasses, in addition to pathological constrictions (spasm), inappropriate vasodilatation, be it more or less than required. Although the signs and symptoms of vascular dysregulation can manifest under baseline conditions, they are observed in particular when the vascular system is challenged.

Dysregulation in anatomically healthy vessels can also occur secondary to other diseases, such as inflammation in remote organs (*secondary vascular dysregulation*, SVD [12])*.* SVD has been described elsewhere [6] and is not part of Flammer syndrome. However, if vascular dysregulation occurs in subjects without causing disease, it is called *primary vascular dysregulation* (PVD) [6]*.* The combination of PVD with a cluster of additional vascular and nonvascular signs and symptoms was previously called PVD syndrome. However, to label this entire condition and to avoid confusion, the term *Flammer syndrome*[1-4] was introduced instead.

### Flammer syndrome

The Greek word ‘syndrome’ means ‘running together’ and denotes in medicine a condition characterized by a set of associated signs and symptoms. In Flammer syndrome, the *primary vascular dysregulation* is tied to a *characteristic physical and psychological condition* and a set of additional *symptoms* and *signs*[1,2,6,8] (Tables 1, 2 and 3). Flammer syndrome occurs in a subset of healthy people. Although this syndrome might even be protective against certain diseases such as arteriosclerosis, it predisposes to other diseases such as normal tension glaucoma, retinal vein occlusion in patients without classical risk factors, or even sudden hearing loss [6]. As a matter of course, the corresponding symptoms or signs can occasionally also be observed in isolated form, independent from the Flammer syndrome.

**Table 1:** Symptoms of Flammer syndrome

**Symptoms of Flammer syndrome**	**Frequency**
Cold hands and/or feet	+++
Long sleep onset time	++
Reduced feeling of thirst	++
Increased sensitivity, e.g.	
Increased response to certain drugs	++
Increased smell sensation	++
Increased pain sensation	+
Increased high altitude sensitivity	+
Increased meteorosensitivity	+
Increased sensitivity to vibration	++
Remarkable assiduousness up to tendency toward perfectionism	++
Tinnitus	+
Muscle cramps	+

+++, often; ++, usually; +, sometimes.

**Table 2:** Signs of Flammer syndrome

**Signs of Flammer syndrome**	**Frequency**
Skin temperature redistribution (distal extremities are colder; trunk is warmer)	+++
Increased skin temperature inhomogeneity under emotional stress (up to reversible red and white skin blotches)	++
Low blood pressure; blood pressure drops especially at night	+++
Low body mass index	++
Nailfold capillaroscopy	
Reduced blood flow velocity	++
Prolonged flow cessation after cold provocation	+++
Hemorrhages	+
Silent myocardial ischemia	+
Altered activity of the autonomic nervous system (beat-to-beat variation)	++
Slightly elevated plasma endothelin-1 level	++
Altered gene expression (measured in lymphocytes)	+++

+++, often; ++, usually; +, sometimes.

**Table 3:** Ocular signs of Flammer syndrome

**Ocular signs of Flammer syndrome**	**Frequency**
In otherwise healthy subjects	
Reduced capacity to autoregulate ocular blood flow	+++
Correlation between the optic nerve head and peripheral blood flow	++
Increased stiffness of retinal vessels	++
Higher spatial irregularities in retinal vessels	++
Reduced neurovascular coupling (endotheliopathy)	+++
Additional signs in patients with normal tension glaucoma	
Optic disc hemorrhages	++
Increased retinal venous pressure	++
Activation of retinal astrocytes	++
Increased blood flow resistance in retroocular vessels	++
Increased oxidative stress	+
Optic nerve compartment syndrome	+
Fluctuating diffuse visual field defects	++

+++, often; ++, usually; +, sometimes.

#### Primary vascular dysregulation

To understand PVD, we will first briefly summarize some aspects of vascular regulation. Since the demand for blood supply in organs or tissues varies greatly over time, sophisticated blood flow (BF) regulation is necessary. The vascular system achieves this on the one hand by adapting perfusion pressure and on the other hand by changing local resistance. The latter is a function of the vascular diameter, which, in turn, is regulated by the tone of the smooth muscle cells in the arteries, veins, and pericytes of the capillaries. These contractile cells receive information from the surrounding tissue, the autonomic nervous system, and particularly from the vascular endothelium [9,13].

Insufficient or improper adaption of BF, despite anatomically healthy vessels and the absence of a causative disease, is termed *primary vascular dysregulation*. The harmless red and white blotches on the face and neck of certain stressed people illustrate the basic characteristics of PVD: The blood supply to a given organ is transiently not correctly adapted to the need of this organ. This can be under- or over-perfusion anywhere in the body. Although in such predisposed people, BF might be normal or only mildly altered under baseline conditions, yet it can drastically change in response to stimuli, such as cold [5] or physical or emotional stress [6].

We only partly know the pathomechanisms of PVD at present. On the one hand, the autonomic nervous system is involved; on the other hand, however, the noninnervated retinal vessels can also be dysregulated, indicating the involvement of other regulatory mechanisms, particularly of the vascular endothelial cells [6].

The vascular endothelium is a thin layer of cells lining the inner parts of arteries, veins, capillaries, and lymphatic vessels, forming a structural barrier between the vessel's lumen and the surrounding tissue. This layer is important for hemostasis, barrier function, immune and inflammatory responses, angiogenesis, and particularly in regulating the vascular tone. In subjects with PVD, the function of the endothelium is changed. However, the endotheliopathy in PVD should not be confused with the endothelial dysfunction observed in diseases such as arteriosclerosis or diabetes.

#### Physical and psychological conditions

Flammer syndrome occurs more often in women than in men, in slender more than in obese subjects, in subjects with systemic hypotension more than in subjects with hypertension, in people with indoor jobs more than in those with outdoor jobs, in academics more than in blue collar workers, and in Asians more often than in Caucasians. Most subjects with Flammer syndrome are physically and mentally particularly active and assiduous, and successful in their jobs. Although these subjects carry the syndrome throughout their lives, the symptoms start to manifest especially during puberty and mitigate with age, in women specifically a few years after menopause. Most subjects with Flammer syndrome indicate that one or both parents suffered from the same syndrome. It is therefore likely an inherited condition.

#### Symptoms

Subjects with Flammer syndrome suffer from a number of symptoms [6] which are summarized in Table 1. The leading symptoms include cold hands and/or feet, low blood pressure, prolonged sleep onset time, shifted circadian rhythm, and reduced feeling of thirst; these subjects are less thirsty but normally drink enough since they are aware of the need to drink. As a rule, these subjects are exceptionally sensitive. This includes aspects such as increased pain sensitivity but also augmented perception of thunderstorms or increased ability to smell, boosted response to high altitude, and increased vibration sensitivity. Sensitivity to certain drugs is also increased (e.g. calcium channel blockers and systemic beta-blockers). Subjects with Flammer syndrome tolerate these drugs well but only at lower doses than usually prescribed. Subjects often conspicuously indicate tinnitus and muscular cramps. If migraineurs suffer from Flammer syndrome, they often experience prodromal symptoms, including visual aura, before the pain attack.

#### Signs

Subjects with Flammer syndrome typically show a number of general (Table 2) and ocular (Table 3) signs [6]. The leading signs include a lower distal temperature (hands, feet, and cornea), while core temperature is normal or even slightly elevated. Although the baseline BF velocity in various organs is either normal or slightly decreased, it drops significantly when triggered. In nailfold capillaroscopy, prolonged blood flow cessation after cold provocation is observed. Blood pressure is generally low or can drop when subjects stand up (orthostatic hypotension) or during sleep (nocturnal over-dipping). As these subjects age, their blood pressure can normalize or even increase above normal.

The level of endothelin-1 in the circulating blood is often slightly increased, and endothelin sensitivity is inversely related to blood pressure. Gene expression in lymphocytes (and probably in other cells as well) is quantitatively altered. The circadian rhythm is delayed by almost 1 h. Analysis of the heart rate variability reveals an autonomic imbalance with sympathetic predominance, and the frequency of silent myocardial ischemia is increased [6]. Under stress, skin temperature becomes more inhomogeneous. This can be observed with the help of thermography and is sometimes so strong that it leads to visible skin blotches.

In the eye (Table 3), the retinal vessels are stiffer, their spatial variability larger, and flow-mediated vasodilation is reduced (manifested as reduced neurovascular coupling). The autoregulatory responses to an intraocular pressure (IOP) increase or blood pressure decrease are reduced or even absent. The altered autoregulation explains why in such cases ocular blood flow correlates with peripheral blood flow. Glaucoma patients with Flammer syndrome have additional signs (Table 3).

### Differentiation from other conditions

Several conditions are similar but not identical to Flammer syndrome [4]. We briefly discuss some of these conditions.

#### Secondary vascular dysregulation

In certain diseases, affected tissue releases vasoactive substances, e.g. endothelin-1, into the circulating blood. This influences remote organs and can lead to vascular dysregulation, particularly in the optic nerve head [9]. The endothelin-1 plasma level is, e.g. often increased in multiple sclerosis, optic neuritis, rheumatoid arthritis, fibromyalgia, or giant cell arteritis [6]. The distinct differences between SVD and PVD have already been described [6].

#### Vegetative dystonia

The clinical picture of this not well-defined condition results from functional disturbances of the autonomic nervous system. Although in Flammer syndrome the autonomic nervous system also plays a role, many symptoms and signs are independent from it. For example, in Flammer syndrome, the retinal vessels are dysregulated, although they are not innervated autonomously.

#### Raynaud's disease

Flammer syndrome should not be confused with Raynaud's disease (RD) [2]. RD is more rare and severe. Patients with RD also respond to cold and psychological stress, but with a more excessive BF decrease. This leads to severe hypoxia of the superficial skin, which turns white and blue. When the BF returns to normal, the skin turns red and throbs or tingles. The loss of BF can cause sores or tissue death. RD may also cause nails to become brittle with longitudinal ridges.

In contrast, the BF disturbance in Flammer syndrome is less acute, less severe, and less localized. It can sometimes lead to pale extremities. However, it does not lead to white fingers or to trophic changes in the extremities. Flammer syndrome comprises many additional vascular and nonvascular symptoms and signs not related to RD [2].

The term Raynaud's phenomenon was coined to describe a similar but weaker entity than Raynaud's disease. Raynaud's phenomenon can also be primary or secondary. The symptoms of Raynaud's phenomenon are to some extent similar to those of PVD, but still more severe and not accompanied with the symptoms and signs of the Flammer syndrome.

#### Migraine

Although subjects with Flammer syndrome suffer more often from migraines (and vice versa), the two entities are not primarily identical and should not be confused [6]. Patients with migraine also often are sensitive, including thermal hypersensitivity between attacks. A migraine attack in a subject with Flammer syndrome often triggers symptoms such as cold hands. A reduction in neurovascular coupling during an acute attack has also been reported, which potentially explains why migraine patients avoid intense light and noises.

Subjects with Flammer syndrome sometimes (but not often) suffer from retinal migraine, also called ‘presumed retinal vasospasm’. A relationship between retinal vasospasm and alterations in the nailfold capillaries was already described in 1939 [6]. The reversible vasoconstriction in the retina indicates that stimuli for vasoconstriction other than the autonomic nervous system must exist. Taking neurovascular coupling into consideration, it seems feasible that the spreading depression acts as a trigger for vasoconstriction [6]. Briefly, migraine and Flammer syndrome have a few aspects in common but also have many distinct differences. Obviously, patients can suffer from both.

### Related diseases

Despite many symptoms and signs, most subjects with Flammer syndrome are healthy people. On the one hand, they may even be relatively protected against certain diseases such as arteriosclerosis or diabetes type 2 since metabolic syndrome occurs less frequently in subjects with low blood pressure, low body mass index, or high physical activity. On the other hand, however, affected subjects have an increased risk of certain other diseases [6], such as normal tension glaucoma [2,8] (see below). Vascular occlusions normally occur in older patients with arteriosclerosis. In subjects with Flammer syndrome, vascular occlusions (including Susac syndrome, anterior ischemic optic neuropathy, and myocardial infarctions) can occur, though rarely, at a young age and in the absence of risk factors for arteriosclerosis. This is particularly true for retinal vein occlusions. Whether some forms of microvascular angina, such as cardiac syndrome X [14] or spasm of coronary arteries such as Prinzmetal angina may be related to Flammer syndrome must be studied since there are similarities. Subjects with Flammer syndrome often report tinnitus and sometimes even sudden hearing loss with mostly good reversibility. Flammer syndrome can be associated with autoimmune diseases such as multiple sclerosis or thyroid dysfunction [6]. Patients with multiple sclerosis often indicate that they had symptoms of Flammer syndrome before they suffered from multiple sclerosis. We hypothesize that very small ischemic lesions in youth may trigger autoimmune diseases that manifest later in life. In micro-infarctions, the dying cells release molecules normally not exposed to the immune system. This may induce an immune response. Flammer syndrome also modifies the phenotype of genetic diseases such as retinitis pigmentosa or Leber's optic neuropathy [6].

#### Normal tension glaucoma

Mild but repeated BF decrease, mainly due to disturbed autoregulation and ocular perfusion pressure fluctuation, leads to an unstable oxygen supply and therefore to an increased local mitochondrial oxidative stress [15-18]. This process is a recognized pathophysiological mechanism of glaucomatous optic neuropathy. Generally, patients who develop glaucomatous damage despite a normal IOP or patients with progressing glaucomatous damage despite well-controlled IOP very often suffer from Flammer syndrome. Although this type of glaucomatous damage is basically the same as in patients with high IOP, there are differences. The retinal venous pressure is on the average higher, splinter hemorrhages occur more often, retinal astrocytes are more often activated, oxidative stress is increased, optic nerve compartment syndrome can more often be detected [6], and the retinal vessels of the optic nerve head are less shifted to the nasal side [1]. Glaucoma patients with Flammer syndrome have particularly large long-term fluctuations of the diffuse component of visual field defects, which is best observed with the help of a Bebie curve (Table 3).

### Diagnostics

Flammer syndrome suspect is based on classical symptoms such as cold hands or feet. Twenty-four-hour blood pressure monitoring helps find arterial hypotension. The diagnosis [6,8,10,19] can then be substantiated by special examinations such as dynamic retinal vessel analysis, nailfold capillaroscopy, or quantification of gene expression in lymphocytes. The gold standard, however, still remains to be defined.

### Treatment

Flammer syndrome is mostly harmless, and therefore, most subjects require no treatment. However, if the symptoms are annoying or affected individuals develop related diseases, we consider treatment as necessary. The intensity of the treatment depends on the clinical picture and the individual situation. Although little research has been conducted on treatment, we can still assist these patients based on the information available. The treatment is based on three pillars: (a) lifestyle management, (b) nutrition, and (c) drug therapy.

#### Lifestyle management

Most subjects with Flammer syndrome know what triggers their vasospastic episodes; as a consequence, one can recommend ways to avoid those. These can include thermal protection, prevention of stress, regular sleep, and regular but not excessive physical activity and moderation in sports. Autogenic training or yoga can help to relax [6]. Enough adaption time should be taken when going to high altitude, etc.

#### Nutrition

The symptoms inversely correlate with body mass index [6]. We therefore recommend eating enough to avoid excessive leanness. Fasting periods trigger symptoms [6]. If blood pressure is low, salt and fluid intake can be increased, particularly in the evening to avoid excessive nocturnal blood pressure dips. Omega-3 fatty acids, as those contained in fish, are also recommended [6]. Since oxidative stress, induced by the unstable oxygen supply, may increase, particularly in the eye, antioxidative diet is considered [6,9,10].

#### Drug treatment

Magnesium [6], a physiological calcium channel blocker (CCB), reduces the vasoconstrictive effect of endothelin-1 and improves BF regulation [6]. A relatively high dose of at least 10–20 mmol/day magnesium is needed. The only side effect observed is diarrhea, which mitigates quickly when the dose is reduced.

If magnesium is not sufficient to improve vascular regulation, a very low dose of CCB [20], preferably nifedipine or amlodipine can be added. It is important to keep the dose low for the following reasons: (1) low doses can have a good effect on regulating vessels, (2) subjects with Flammer syndrome have increased drug sensitivity, and (3) in most cases, a further decrease blood pressure is not desirable. Before and after CCB therapy is initiated, blood pressure should be controlled; CCB treatment was recently reviewed [20]. The combination of magnesium with low-dose CCB not only improves regulation of blood vessels as observed with the Dynamic Retinal Vessel Analyzer [7] but also reduces retinal venous pressure and optic nerve compartmentalization [6].

To protect the mitochondria from oxidative stress, ginkgo biloba [6] can be used at the dose of 120 mg extract per day. In case of severe arterial hypotension, low doses of fludrocortisone [6] (0.1 mg twice per week) can be effective; mineralocorticoids have fewer side effects than glucocorticoids. Vasoconstrictive drugs should be avoided.

## Conclusions

Although Flammer syndrome is quite prevalent and mostly benign, it may contribute to the occurrence and progression of potentially serious diseases such as normal tension glaucoma. At present, we are still in the early stage of research in this field. The authors suggest the following steps in order to promote research area:

1. The phenotype should be further characterized, qualitatively and quantitatively.

2. The underlying pathophysiology should be studied in more detail, particularly the role of genetics, the autonomic nerve system, the vascular endothelial cells, and the mitochondria.

3. The role of environmental factors such as light exposure or nutrition needs to be established.

4. The relationship between vascular factors and autoimmune diseases should be studied.

5. The cause of the combination of vascular dysregulation with nonvascular signs and symptoms needs to be found.

6. The statistical and causal relationships with related diseases should be established.

7. The gold standard for diagnosis must be defined.

8. The age-, gender-, and race-dependent prevalence should be studied.

The present treatment modalities are of limited value and not yet based on well-controlled studies. Vitamin D, for example, may play a role but has not been studied yet. Developing novel drug targets and more efficient treatment approaches is desirable [21]. Establishing the risk for related diseases may lead to predictive and preventive diagnostics, and treatment tailored to the person.

## Abbreviations

BF: Blood flow; CCB: Calcium channel blocker; IOP: Intraocular pressure; PVD: Primary vascular dysregulation; RD: Raynaud's disease; SVD: Secondary vascular dysregulation.

## Competing interests

The authors declare that they have no competing interests.

## Authors' contributions

KK wrote the manuscript and all coauthors read and corrected the first version. All authors read and approved the final manuscript.

## Authors' information

Dr. KK is a glaucoma specialist from the Department of Ophthalmology, University of Basel, Switzerland. Prof. RR is Shelley and Steven Einhorn Distinguished Chair and is the Surgeon Director and Chief of the Glaucoma Services, Einhorn Clinical Research Center, New York Eye and Ear Infirmary of Mount Sinai, New York, NY, USA. He is also the Founder, Medical Director, and Chairman in the Scientific Advisory Board of the Glaucoma Foundation. CET is Professor and Chairman, Clinica Oculistica, Di.N.O.G.M.I. Univestity of Genova, IRCCS Azienda Ospedaliera Universitaria San Martino – IST, Genova, Italy. Prof. DMK is a glaucoma specialist from the Department of Ophthalmology, Seoul National University, Seoul, Korea. Prof. MSK is a glaucoma specialist from the Department of Ophthalmology, University of Ulsan, Seoul, Korea. Prof. AG is the Chief of Internal Medicine and Cardiology Department in Ospedale San Giovanni, Bellinzona and is a Professor at the University of Zürich, Switzerland. Prof. OG is a Secretary-General of the European Association for Predictive, Preventive and Personalised Medicine (with special interest in molecular diagnostics) and is from the Department of Radiology, Friedrich-Wilhelms-University of Bonn, Germany. Prof. CE is a glaucoma specialist and the Medical Director of Eye Clinic Wittenbergplatz, Berlin, Germany. Prof. HAR is the Head of Glaucoma Department and Head of Fuchs Laboratory, Department of Ophthalmology, SALK/Paracelsus Medical University, Salzburg, Austria. Dr. TK is an ophthalmologist with special interest in ocular blood flow and medical retina, Ophthalmology Department, Osaka Medical College, Takatsuki, Japan. Prof. NK is the Head of the Diagnostic and Glaucoma Department, University of Medical and Biological Agency of Russian Federation, Moscow, Russian Federation. Prof. KY is the President-elect of the Chinese Ophthalmological Society and Chief of the Eye Center of Second Affiliated Hospital, Zhejiang University College of Medicine, Hangzhou, China (special interests are cataract and glaucoma).
